# Galectin-1 stimulates motility of human umbilical cord blood-derived mesenchymal stem cells by downregulation of smad2/3-dependent collagen 3/5 and upregulation of NF-*κ*B-dependent fibronectin/laminin 5 expression

**DOI:** 10.1038/cddis.2014.3

**Published:** 2014-02-06

**Authors:** S P Yun, S-J Lee, Y H Jung, H J Han

**Affiliations:** 1 Department of Veterinary Physiology, College of Veterinary Medicine, Research Institute for Veterinary Science, Seoul National University, Seoul, Korea; 2 BK21 PLUS Creative Veterinary Research Center, Seoul National University, Seoul, Korea

**Keywords:** umbilical cord blood-derived mesenchymal stem cells, galectin-1, extracellular matrix proteins, motility

## Abstract

Galectin-1 (Gal-1) belongs to a family of endogenous lectins with conserved carbohydrate recognition domains binding *β*-galactosidase sugars and plays a vital role in regulating stem cell functions including determination of cell fate. However, our understanding of the functional roles of Gal-1 in human umbilical cord blood-derived mesenchymal stem cells (UCB-MSCs) is still fragmentary and incomplete. Gal-1 significantly increased motility after a 24-h incubation, and this effect was inhibited by *β*-lactose. We analyzed 17 extracellular matrix (ECM) genes in UCB-MSCs. Gal-1 decreased the expression of collagen genes *COL3A1* (*COL-3*) and *COL5A1* (*COL-5*) but increased the expression of fibronectin (FN) and laminin 5 (LM-5), that were reversed by *β*-lactose. Gal-1 increased protein kinase C (PKC), c-Src, and caveolin-1 (Cav-1) phosphorylation that was attenuated by *β*-lactose and the Src inhibitor PP2. In addition, pretreatment with the lipid raft disruptor M*β*-CD and the PKC inhibitors inhibited Gal-1-induced UCB-MSC motility. In addition, Gal-1 reduced smad2/3 phosphorylation and induced nuclear factor (NF)-*κ*B phosphorylation. Pretreatment with M*β*-CD attenuated Gal-1-reduced smad2/3 phosphorylation, COL-3, and COL-5 expression but did not affect NF-*κ*B phosphorylation, FN, or LM-5 expression. In contrast, PKC inhibitors only attenuated NF-*κ*B phosphorylation, FN, and LM-5 expression. Reconstructing Gal-1-induced genetic changes by replacing it with siRNA specific for *COL-3* or *COL-5*, or treatment of the cells with FN and LM-5 proteins, increased motility and its related proteins such as focal adhesion kinase, Akt, Erk, integrins, and matrix metalloproteinase-2. A combined treatment with *COL-3*/*COL-5* siRNA or FN/LM-5 compared with that of single treatments was synergistic. However, a single Gal-1 treatment maximally stimulated motility and related protein phosphorylation/expression. These results demonstrate that Gal-1 stimulated human UCB-MSC motility by decreasing COL-3/COL-5 expression and increasing FN/LM-5 expression through a PKC-dependent NF-*κ*B and c-Src/Cav-1-dependent smad2/3 pathway that was critical for governing the activation of FAK, Akt, Erk, integrins, and MMP2.

Cell motility is regulated by a complex network of microenvironments and extracellular factors including carbohydrate-binding proteins.^1^ One of these carbohydrate-binding proteins is galectin (Gal) that has affinity for *β*-galactoside-containing glycoconjugates.^2, 3^ To date, 10 of the 15 mammalian members have been identified in humans,^4^ and all can be classified according to their carbohydrate recognition domains.^2^ Among them, Gal-1 is expressed by various stem cells, including embryonic, hematopoietic, neural, and keratinocyte stem cells^3, 5, 6^ and regulates various stem cell functions such as tumor promotion and differentiation.^7^ We and others have reported that Gal-1 is expressed on mouse embryonic stem cells (ESCs)/adult neural stem cells (NSC) and promotes their proliferation through its carbohydrate-binding ability.^3, 8^ In addition, Gal-1 is a critical factor in the function of mouse mesenchymal stromal cell-mediated tumor promotion and invasion.^7^ Until now, little research has been conducted on the function of Gal-1 in umbilical cord blood-derived mesenchymal stem cells (UCB-MSCs), although UCB-MSCs have reportedly more abundant expression of the Gal-1 gene (*LGALS1*) than the bone marrow-derived hMSCs.^9^

Recent studies clearly implicate the functional relevance of Gal-1 and extracellular matrix (ECM) proteins in mediating wound healing, motility, and adhesion.^10^ Interestingly, alterations in ECM protein expression play a contrasting role in stem cell function. Treating ESCs and MSCs with collagen-1 (COL-1), fibronectin (FN), and laminin-111 (LM-111) stimulates proliferation and migration.^11, 12, 13, 14^ In contrast, decreased COL-1 and LM-5 expression stimulates motility and differentiation and/or leads to programmed cell death through upregulation of matrix metalloproteinase (MMP) expression in fibroblasts and mammary epithelial cells.^15, 16^ In this regard, it is possible that Gal-1 stimulates UCB-MSC motility through up- or down-regulation of ECM protein expression. Yet, despite the absence of the role of Gal-1-related ECM proteins during stem cell functions for the current molecular cell biology paradigm, a number of independent experiments link ECM proteins to normal growth control, differentiation, and motility.^10, 13, 16^ In addition, ECM proteins are a downstream target of the key transcription factors NF-*κ*B (nuclear factor *κ*-light-chain-enhancer of activated B cells) and smad.^17, 18^ However, no evidence indicates a direct correlation between Gal-1-related ECM protein expression and motility alterations in human UCB-MSCs, although the function and regulation of ECM proteins has been investigated in various cell types including stem cells. Thus, it was considered important to clarify the Gal-1-related ECM protein responses when considering UCB-MSCs for regeneration strategies, and in particular we aimed to understand the molecular mechanisms involved in galactosidase-binding lectin-mediated MSC motility.

Human UCB-MSCs derived from cord blood after birth have self-renewal capacity and can differentiate into multiple cell types.^19^ Thus, human UCB-MSCs might be regarded as a versatile biological system, and their use has led to major advances in cell therapy and regeneration strategies in bone regeneration and spinal cord injury.^20, 21^ Gal-1 and ECM proteins have relevance to stabilize stem cell transplantation, regulate motility, and control differentiation.^22, 23, 24^ Although several clinical studies have detailed stem cell transplantation and/or therapeutic methods using Gal-1 and ECM proteins,^23, 24^ serious problems such as degeneration of scaffold protein, destruction of stem cell constituents, and alterations in stem cell functions may arise during transplantation and cell therapy.^25^ A prerequisite for effective clinical application is selecting high-quality input materials, as well as understanding the regulatory mechanisms mediating various processes such as motility. If we assume that exogenous Gal-1 regulates ECM proteins and improves subsequent motility, the downstream signaling mechanisms involved may present promising targets for stem cell therapy. Thus, determining how Gal-1 and signal transduction proteins function as an ensemble to regulate motility remains a major challenge. Therefore, we examined the involvement of ECM proteins in Gal-1-induced human UCB-MSC motility and its related signal pathways.

## Results

### Effect of Gal-1 on human UCB-MSC motility and ECM protein expression

**Figure 1:**
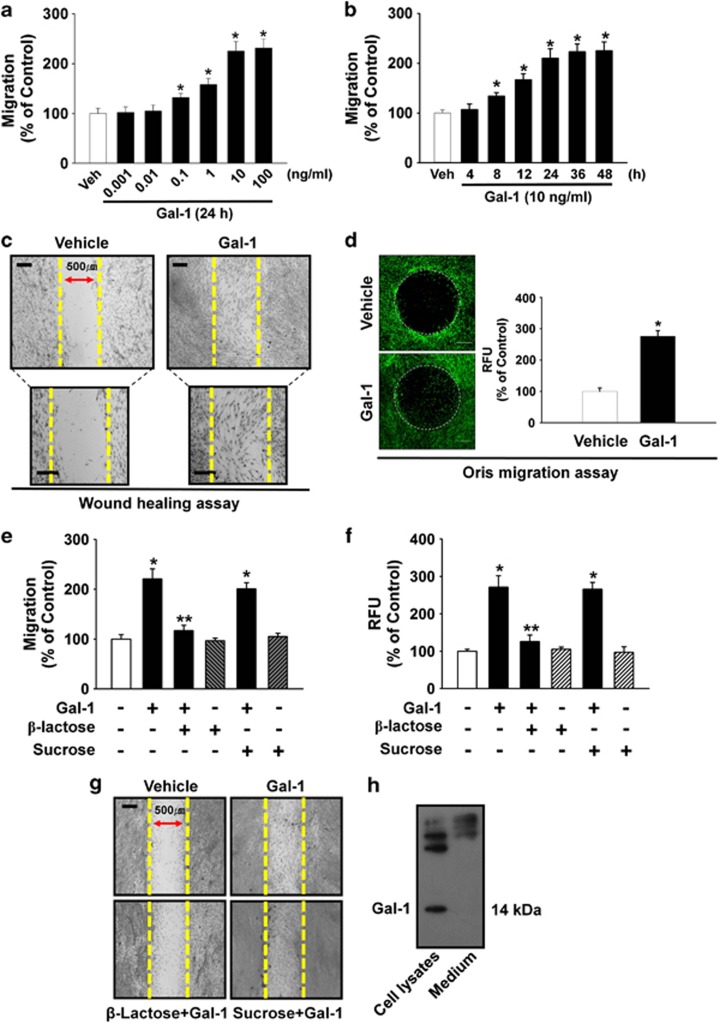
Effects of Gal-1 on human UCB-MSC motility. Dose (**a**) and time (**b**) response of Gal-1 in a Boyden chamber motility assay. Cells were treated with different doses of Gal-1 (0–100 ng/ml) for 24 h or with 10 ng/ml Gal-1 for various times (0–48 h), and cells that had migrated to the lower surface of the filter were enumerated. A minimum of 100 cells was counted per sample. Values represent the mean±S.E. of five independent experiments with triplicate dishes. *P<0.05 *versus* vehicle. (**c**) *In vitro* human UCB-MSC wound healing migration assay in the absence and presence 10 ng/ml of Gal-1. Ten fields per plate were examined. Scale bars represent 100 *μ*m (magnification × 100) in top panel. Scale bars represent 100 *μ*m (magnification × 200) in bottom panel. (**d**) Oris cell motility assay. Cells were treated with Gal-1 (10 ng/ml) for 24 h and stained with calcein AM (green, left panel). Dot circles indicate analytical zone of a plate reader for quantification of fluorescence (*n*=3). Scale bars represent 200 *μ*m (magnification × 40). Fluorescence in the analytical zone was quantified with a plate reader (right panel). Data represent means±S.E. of five independent experiments with triplicate dishes. *P<0.05 *versus* vehicle. (**e**) The cells were pretreated with *β*-lactose (10 mM) or sucrose (10 mM; used as an osmolarity control) for 30 min before 10 ng/ml Gal-1 treatment for 24 h. Cells that had migrated to the lower surface of the filter were enumerated. A minimum of 100 cells were counted per sample. The values represent the mean±S.E. of four independent experiments with triplicate dishes. *P<0.05 *versus* control, **P<0.05 *versus* Gal-1 alone. (**f**) Oris cell motility assay. Cells were pretreated with *β*-lactose or sucrose for 30 min before 10 ng/ml Gal-1 treatment for 24 h and stained with calcein AM (5 *μ*M). Fluorescence in the analytical zone was quantified with a plate reader. Data represent means±S.E. of five independent experiments with triplicate dishes. *P<0.05 *versus* control, **P<0.05 *versus* Gal-1 alone. (**g**) *In vitro* human UCB-MSC wound healing motility assay. Ten fields per plate were examined. Scale bars represent 100 *μ*m (magnification × 100). (**h**) The cells were cultured in serum-free medium for 24 h, and then Gal-1 was detected from total cell lysates or cell culture medium by TCA precipitation and western blot analysis as described in the ‘Materials and Methods'. Each example shown is representative of five experiments. Gal-1, galectin-1; UCB-MSC, umbilical cord blood-derived mesenchymal stem cells; Veh, vehicle

The cells were incubated with various concentrations (0–100 ng/ml) of Gal-1. As shown in Figure 1a, increasing the concentration of Gal-1 from 0.1 to 100 ng/ml induced cell motility in a dose-dependent manner. In addition, an increase in cell motility was observed after an 8-h incubation with 10 ng/ml Gal-1 (Figure 1b). We further explored the ability of 10 ng/ml Gal-1 to induce cell motility for 24 h using an *in vitro* wound healing migration assay and the Oris cell migration assay (Platypus Technologies, Madison, WI, USA). In contrast to the control, Gal-1 evoked substantial migration of cells into the denuded area (Figure 1c). These results were confirmed by increased motility of Gal-1-stimulated cells directly in the Oris cell migration assay (Figure 1d). In addition, Gal-1 (10 ng/ml) significantly induced the motility of human adipose-derived mesenchymal stem cells (AD-MSCs) (32% increase compared with the control; *P<*0.01), although the effect of Gal-1 in AD-MSC motility was lower than in UCB-MSCs (125% increase compared with the control; *P*<0.01) (Supplementary Data 1). These results suggest that the functional role of Gal-1 to induce the motility is reproducible in other types of MSCs.

To examine the specificity of Gal-1 in UCB-MSC motility, we tested whether Gal-3 could induce motility in UCB-MSCs. We found that Gal-3 also stimulated cell migration (48.3% above control; *P*<0.01), but it was less potent compared with Gal-1 (125% above control; *P*<0.01) (Supplementary Data 2). The cells were treated with Gal-1 either in the presence or absence of *β*-lactose (10 mM) or sucrose (10 mM; used as an osmolarity control) for 24 h to determine Gal-1 involvement in carbohydrate-binding activity. The Gal-1-induced increase in cell motility was attenuated by *β*-lactose (Figures 1e–g). In addition, the effect of intrinsic Gal-1 on cell motility was examined. As shown in Figure 1h, Gal-1 was expressed in human UCB-MSCs but was not detected in culture media. Knockdown of intrinsic Gal-1 with Gal-1 small interfering RNA (siRNA) significantly attenuated the motility of UCB-MSCs induced by Gal-1 treatment (59.7% decrease compared with the Gal-1 treatment; *P*<0.05) (Supplementary Data 3a and b). However, Gal-1 treatment did not enhance the level of intrinsic Gal-1 expression (Supplementary Data 3c). In addition, mitomycin C (1 *μ*g/ml), a cell cycle arrest compound, partially inhibited Gal-1-induced cell motility (Supplementary Data 4).

**Figure 2:**
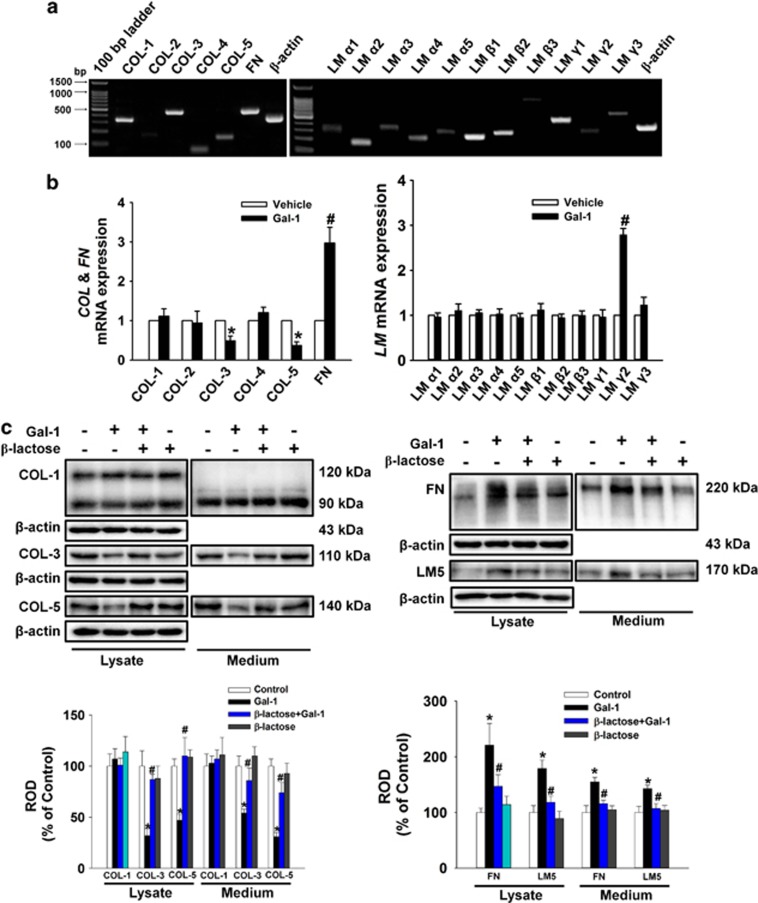
Effects of Gal-1 on ECM protein expression. (**a**) Total RNA from cells was reverse transcribed, and ECM cDNAs were amplified by PCR as described in the Materials and Methods. The example shown is representative of five independent experiments. (**b**) Cells were treated with 10 ng/ml Gal-1 for 12 h. The mRNA expression of ECM components was measured in cells using real-time PCR as described in the Materials and Methods. The data are reported as the mean±S.E. of four independent experiments, each conducted in triplicate. *P<0.05 *versus* vehicle. (**c**) The cells were pretreated with *β*-lactose (10 mM) for 30 min before 10 ng/ml Gal-1 treatment for 24 h. Total protein was extracted and blotted with COL-1, COL-3, COL-5, FN, and LM-5 antibody. Each example shown is representative of five independent experiments. The lower or right part of (**c**) depicting the bars denotes the mean±S.E. of five independent experiments for each condition determined by densitometry relative to *β*-actin. *P<0.05 *versus* control, ^*#*^*P*<0.05 *versus* Gal-1 alone. COL, collagen; ECM, extracellular matrix; FN, fibronectin; LM, laminin; ROD, relative optical density

In experiments to determine the existence of ECM component types in human UCB-MSCs, we detected signals for amplicons of *COL*, *FN*, and *LM* mRNAs (Figure 2a). Gal-1 reduced *COL3A1* (*COL-3*) and *COL5A1* (*COL-5*) mRNA expression levels but increased *FN1* (*FN*) and *LAMC2* (*LM-γ2*) mRNA expression levels (Figure 2b). Consistent with these results, Gal-1 decreased the protein expression of COL-3 and COL-5 but increased FN and LM-5 in total lysates and medium, and this was reversed by *β*-lactose (Figure 2c). These results suggest that Gal-1 stimulation of human UCB-MSC motility may be mediated via regulation of ECM protein expression.

### Effect of Gal-1 on protein kinase C (PKC) activation and c-Src dependent caveolin-1 (Cav-1) activation

**Figure 3:**
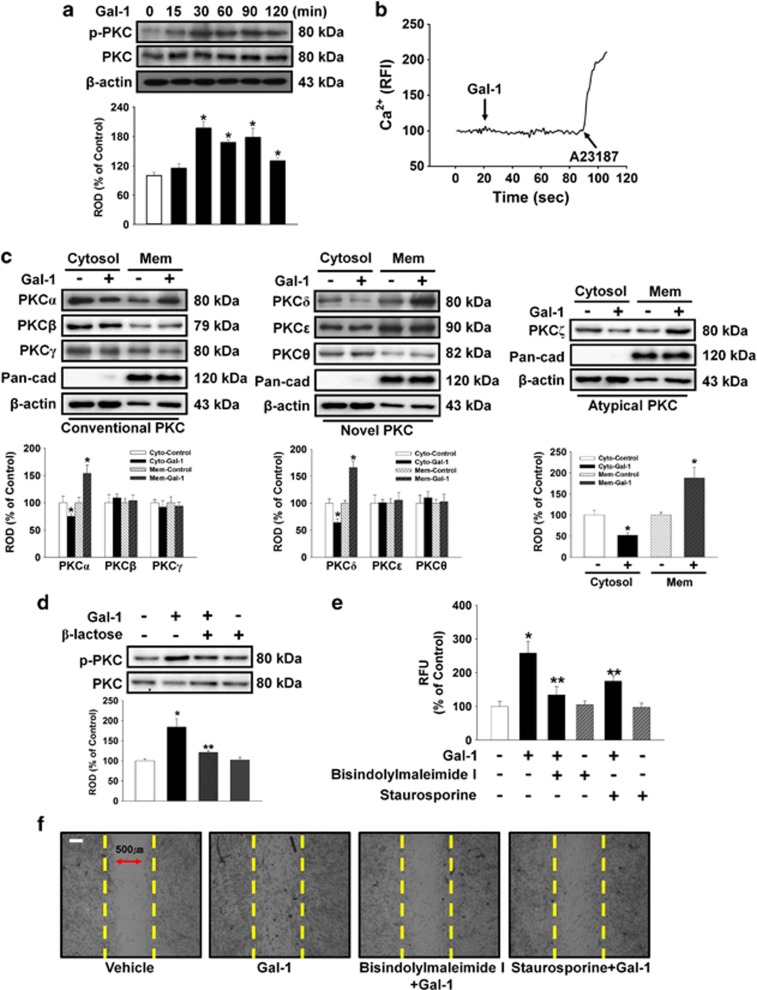
Involvement of PKC phosphorylation and translocation. (**a**) The cells were incubated in the presence of Gal-1 (10 ng/ml) for varying periods of time (0–120 min) and then harvested. Total protein was extracted and blotted with phospho-PKC and total-PKC antibody. (**b**) The cells were incubated with 2 *μ*M Fluo-3/AM in serum-free medium for 40 min and treated with Gal-1 (10 ng/ml) and were then treated with A23187 (10 *μ*M, Ca^2+^ ionophore). Gal-1 and A23187-induced Ca^2+^ influx was measured. Changes in [Ca^2+^]_i_ were monitored by confocal microscopy, and data are expressed as relative fluorescence intensity (F/F0%, arbitrary unit). (**c**) The cells were stimulated with Gal-1 (10 ng/ml) for 90 min, and extracted cytosolic and membrane proteins were then detected with PKC translocation of PKC isoforms. Western blot analysis showed that Gal-1 induced the membrane translocation of PKC*α*, *δ*, and *ζ*. (**d**) The cells were pretreated with *β*-lactose (10 mM) for 30 min before 10 ng/ml Gal-1 treatment for 90 min. Total protein was extracted and blotted with phospho- and total PKC antibody. (**a**, **c**, and **d**) Each example shown is representative of four independent experiments. The lower or right part of (**a**, **c**, and **d**) depicting the bars denote the mean±S.E. of four independent experiments for each condition determined by densitometry relative to *β*-actin or total-PKC. *P<0.05 *versus* control, **P<0.05 *versus* Gal-1 alone. (**e**) Oris cell motility assay. Cells were pretreated with 10 *μ*M bisindolylmaleimide I and 10 *μ*M staurosporine (PKC inhibitors) for 30 min before 10 ng/ml Gal-1 treatment for 24 h and stained with calcein AM (5 *μ*M). Fluorescence in the analytical zone was quantified with a plate reader. Data represent means±S.E. of five independent experiments with triplicate dishes. *P<0.05 *versus* control, **P<0.05 *versus* Gal-1 alone. (**f**) *In vitro* UCB-MSC wound healing motility assay. Ten fields per plate were examined. Scale bars represent 100 *μ*m (magnification × 100). Cytosol, cytosolic; Mem, membrane; PKC, protein kinase C; ROD, relative optical density

This experiment assessed whether Gal-1 induced phosphorylation and translocation of PKC in human UCB-MSCs to examine involvement of the PKC pathway in Gal-1-induced cell motility. Gal-1 increased PKC phosphorylation from 15 to 120 min (Figure 3a), but did not affect calcium influx (Figure 3b). In addition, translocation of PKC*α*, PKC*δ*, and PKC*ζ* from the cytosol to the membrane compartment was observed after cells were treated with 10 ng/ml Gal-1 for 90 min (Figure 3c). *β*-Lactose reduced Gal-1-induced PKC phosphorylation (Figure 3d). To further elucidate the involvement of PKC in Gal-1-induced cell motility, the cells were pretreated with bisindolylmaleimide I and staurosporine before Gal-1 treatment. As shown in Figures 3e and f, bisindolylmaleimide I and staurosporine reduced the Gal-1-induced increase in cell motility.

**Figure 4:**
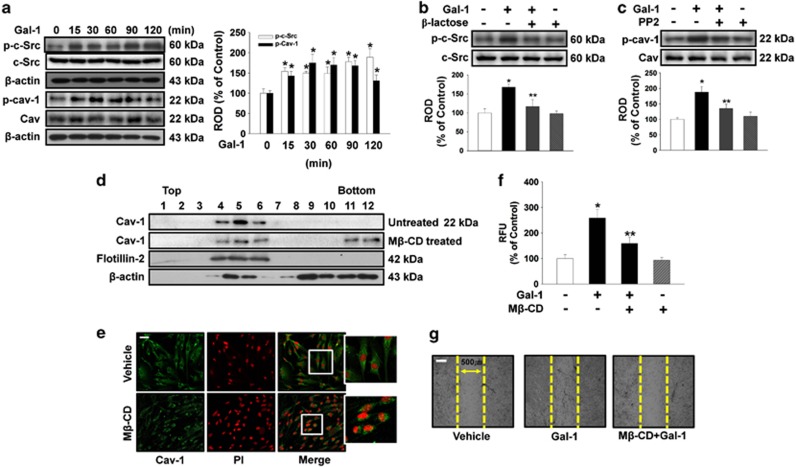
Involvement of c-Src and Cav-1 in effect of Gal-1. (**a**) The cells were incubated in the presence of Gal-1 (10 ng/ml) for varying periods of time (0–120 min) and the phosphorylation of c-Src and Cav-1 was detected by western blot analysis. (**b**) The cells were pretreated with *β*-lactose (10 mM) for 30 min before 10 ng/ml Gal-1 treatment for 90 min. Total protein was extracted and blotted with phospho-c-Src antibody. (**c**) The cells were pretreated with PP2 (10 *μ*M) for 30 min before 10 ng/ml Gal-1 treatment for 90 min. Total protein was extracted and blotted with phospho-Cav-1 antibody. (**d**) Lysates of untreated (control) or lipid raft disruptor 10 mM M*β*-CD-treated cells were subjected to discontinuous sucrose density gradient fractionation. Each fraction was assessed by western blot analysis. (**a**–**d**) Each example shown is representative of four independent experiments. The lower and right part of (**a**–**c**) depicting the bars denote the mean±S.E. of four independent experiments for each condition determined by densitometry relative to *β*-actin, c-Src, or Cav. *P<0.05 *versus* control, **P<0.05 *versus* Gal-1 alone. (**e**) Control and 10 mM M*β*-CD-treated cells were immunostained for Cav-1 (green), and each nucleus was stained with propidium iodide (red). Each example shown is representative of three experiments. Scale bars represent 100 *μ*m (magnification × 100). (**f**) Oris cell motility assay. Cells were pretreated with M*β*-CD (10 mM) for 30 min before 10 ng/ml Gal-1 treatment for 24 h and stained with calcein AM (5 *μ*M). Fluorescence in the analytical zone was quantified with a plate reader. Data represent means±S.E. of five independent experiments with triplicate dishes. *P<0.05 *versus* control or vehicle, **P<0.05 *versus* Gal-1 alone. (**g**) *In vitro* UCB-MSC wound healing motility assay. Ten fields per plate were examined. Scale bars represent 100 *μ*m (magnification × 100). Cav-1, caveolin-1; M*β*-CD, methyl-*β*-cyclodextrin; ROD, relative optical density

The effect of Gal-1 on phosphorylation of c-Src proteins, which are believed to be essential factors in Cav-1 activation, was examined to confirm the effect of Gal-1 on cell motility. Gal-1 increased phosphorylation of c-Src and Cav-1 from 15 to 120 min (Figure 4a) that was inhibited by *β*-lactose (Figure 4b). In addition, the c-Src inhibitor PP2 reduced Gal-1-induced phosphorylation of Cav-1 (Figure 4c). These results establish Gal-1 as an inducer of Cav-1 phosphorylation in human UCB-MSCs. As Cav-1 directly binds to cholesterol, the most important cofactor in caveolar morphogenesis, we first examined the effect of methyl-*β*-cyclodextrin (M*β*-CD, lipid raft disruptor) on caveolar structure to confirm the structural importance of membrane rafts in Cav-1-dependent signaling. Using discontinuous sucrose density gradient fractionation, we confirmed that the Cav-enriched membrane fraction (Figure 4d, fractions 4–6) decreased after treatment with 10 mM M*β*-CD for 1 h (Figure 4d). In addition, caveolar organization was disrupted when cholesterol was depleted from the membrane by means of M*β*-CD (Figure 4e). In addition, pretreatment with M*β*-CD significantly blocked the Gal-1-induced increase in cell motility (Figures 4f and g).

### Effect of Gal-1 on smad2/3 and NF-*κ*B phosphorylation

**Figure 5:**
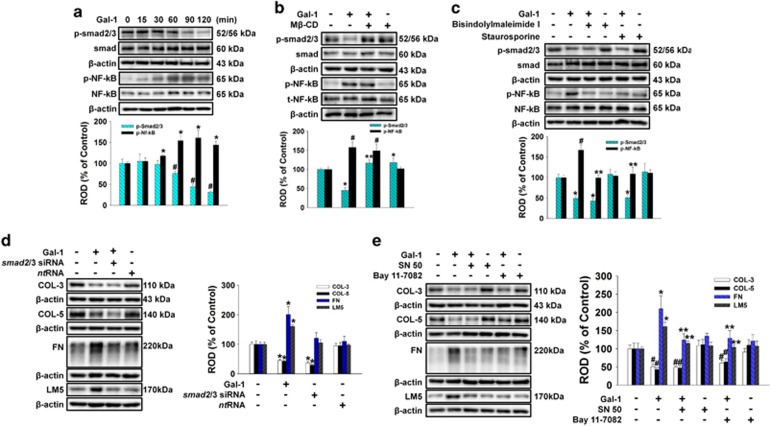
The smad2/3 and NF-*κ*B activation by Gal-1 regulated ECM protein expression. (**a**) The cells were incubated in the presence of Gal-1 (10 ng/ml) for varying periods of time (0–120 min) and phosphorylation of smad2/3 and NF-*κ*B was detected by western blot analysis. (**b**) The cells were pretreated with M*β*-CD (10 mM) for 30 min before 10 ng/ml Gal-1 treatment for 120 min. Total protein was extracted and blotted with phospho-smad2/3 and phospho-NF-*κ*B antibody. (**c**) The cells were pretreated with bisindolylmaleimide I (10 *μ*M) and staurosporine (10 *μ*M) for 30 min before 10 ng/ml Gal-1 treatment for 120 min. Total protein was extracted and blotted with phospho-smad2/3 and phospho-NF-*κ*B antibody. (**d**) The cells were pretreated with NF-*κ*B inhibitors SN 50 (500 ng/ml) and bay 11-7082 (10 *μ*M) for 30 min before 10 ng/ml Gal-1 treatment for 24 h. Total protein was extracted and blotted with COL-3, COL-5, FN, and LM-5 antibody. (**e**) Cells were transfected for 24 h with either *smad2/3-*specific siRNA (200 pmol/l) or *non-targeting* control siRNA (200 pmol/l) using Hyperfectamine or Gal-1 single treatment. Total protein was extracted and blotted with COL-3, COL-5, FN, and LM-5 antibody. (**a–e**) Each example shown is representative of five independent experiments. The lower and right part of (**a**–**e**) depicting the bars denote the mean±S.E. of five independent experiments for each condition determined by densitometry relative to *β*-actin. *^,#^*P*<0.05 *versus* control, **P<0.05 *versus* Gal-1 alone. NF-*κ*B, nuclear factor *κ*-light-chain-enhancer of activated B cells; ROD, relative optical density

To examine the role of smad2/3 and NF-*κ*B in Gal-1-regulated ECM protein expression, we determined whether Gal-1 affected phosphorylation of smad2/3 and NF-*κ*B in human UCB-MSCs. As shown in Figure 5a, smad2/3 phosphorylation decreased from 60 to 120 min after 10 ng/ml Gal-1 treatment, but NF-*κ*B phosphorylation increased from 30 to 120 min. In addition, pretreatment with M*β*-CD significantly blocked Gal-1-reduced phosphorylation of smad2/3, but no effect was observed on NF-*κ*B phosphorylation (Figure 5b). In contrast, the PKC inhibitors bisindolylmaleimide and staurosporine inhibited Gal-1-induced phosphorylation of NF-*κ*B but did not affect smad2/3 phosphorylation (Figure 5c). To further elucidate the involvement of smad2/3 in Gal-1-related ECM component protein expression, cells were transfected with *smad2/3* siRNA or nontargeting siRNA before Gal-1 treatment. Consistent with the effect of Gal-1, only *Smad2/3* siRNA inhibited the expression of COL-3 and COL-5 (Figure 5d). In addition, the NF-*κ*B inhibitors SN 50 and bay 11-7082 inhibited Gal-1-induced FN and LM-5 expression but did not affect COL-3 or COL-5 expression (Figure 5e).

### Role of Gal-1-related COL-3, COL-5, FN, and LM-5 in human UCM-MSC motility

**Figure 6:**
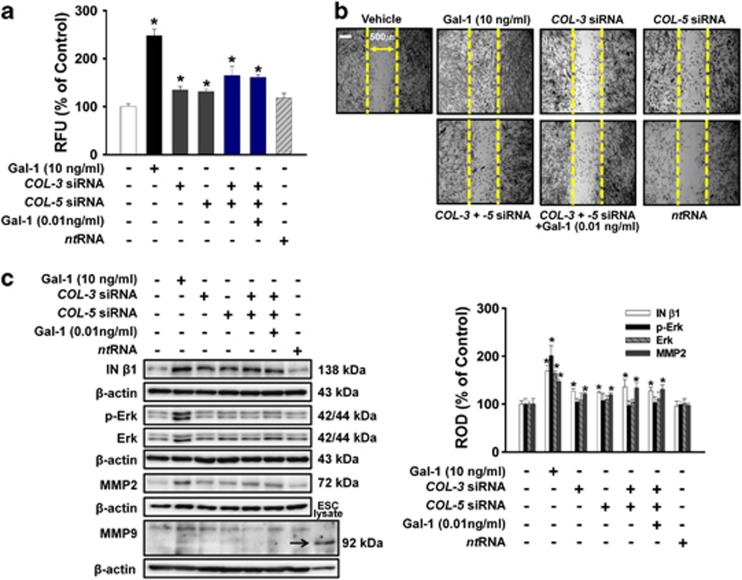
Effects of Gal-1-reduced COL-3 and COL-5 on UCB-MSC motility. Cells were pretreated with *COL-3* siRNA, *COL-5* siRNA, combination of *COL-3* and *COL-5* siRNA, combination of *COL-3*, *COL-5* siRNA, and Gal-1 (0.01 ng/ml), and *nontargeting* control siRNA for 30 min or 6 h before with or without Gal-1 (10 ng/ml) exposure for 24 h. (**a**) Oris cell motility assay. Fluorescence in the analytical zone was quantified with a plate reader. Data represent means±S.E. of five independent experiments with triplicate dishes. *P<0.05 *versus* control. (**b**) *In vitro* UCB-MSC wound healing motility assay. Ten fields per plate were examined. Scale bars represent 100 *μ*m (magnification × 100). (**c**) Cells were pretreated with *COL-3* siRNA, *COL-5* siRNA, combination of *COL-3* and *COL-5* siRNA, combination of *COL-3* siRNA, *COL-5* siRNA, and Gal-1 (0.01 ng/ml), and *nontargeting* control siRNA for 30 min or 6 h before with or without Gal-1 (10 ng/ml) for 24 h. Total protein was extracted and blotted with MMP2, MMP9, phospho-Erk, total-Erk, and IN-*β*1 antibody. ESC lysate is positive control of MMP9.^59^ The right part of (**c**) depicting the bars denotes the mean±S.E. of five independent experiments for each condition determined by densitometry relative to *β*-actin. *P<0.05 *versus* control. COL, collagen; MMP, matrix metalloproteinase; ROD, relative optical density

To investigate the involvement of COLs in Gal-1-mediated UCB-MSC motility, the cells were treated with Gal-1 (10 ng/ml), *COL-3* siRNA*, COL-5* siRNA, the combination of *COL-3* and *COL-5* siRNA, and the combination of *COL-3*, *COL-5* siRNA, and Gal-1 (0.01 ng/ml). As shown in Figures 6a and b, all treatments increased cell motility. Notably, the combination of *COL-3-* and *COL-5-*specific siRNAs (65% increase compared with the control; *P*<0.05) increased cell motility more than *COL-3* (34% increase compared with the control; *P*<0.05) or *COL-5* (30% increase compared with the control; *P*<0.05) single siRNA treatment, and the combination of *COL-3* and *COL-5* siRNAs had a synergistic effect on cell motility (Figures 6a and b). In addition, treatment with Gal-1 (10 ng/ml), *COL-3* siRNA, *COL-5* siRNA, the combination of *COL-3* and *COL-5* siRNA, and the combination of *COL-3*, *COL-5* siRNA, and Gal-1 (0.01 ng/ml) increased phosphorylation and/or expression of MMP2, and integrin (IN)-*β*1, but Gal-1 only increased Erk phosphorylation and expression. We could not detect MMP9 in human UCB-MSCs (Figure 6c).

**Figure 7:**
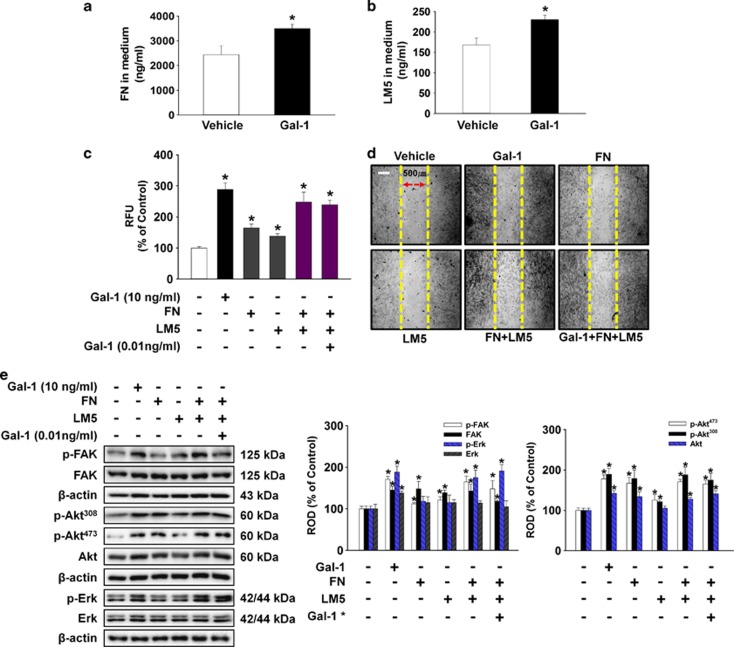
Effects of Gal-1-induced release of FN and LM-5 on UCB-MSC motility. (**a** and **b**) The cells were treated with 10 ng/ml Gal-1 for 24 h; FN and LM-5 production was then detected. Values represent means±S.E. of three independent experiments with triplicate dishes. *P<0.05 *versus* control. (**c**–**e**) Cells were treated with Gal-1 (10 ng/ml), FN (700 ng/ml), LM-5 (62.5 ng/ml), combination of FN and LM-5, and combination of FN, LM-5, and Gal-1 (0.01 ng/ml) for 24 h. (**c**) Oris cell motility assay. Fluorescence in the analytical zone was quantified with a plate reader. Data represent means±S.E. of five independent experiments with triplicate dishes. *P<0.05 *versus* control. (**d**) *In vitro* UCB-MSC wound healing motility assay. Ten fields per plate were examined. Scale bars represent 100 *μ*m (magnification × 100). (**e**) Total protein was extracted and blotted with phospho-FAK, FAK, phospho-Akt^thr308^, phospho-Akt^ser473^, Akt, phospho-Erk, and Erk antibody. The right part of (**e**) depicting the bars denotes the mean±S.E. of five independent experiments for each condition determined by densitometry relative to *β*-actin. *P<0.05 *versus* control. Gal-1* indicates the 0.01 ng/ml concentration of Gal-1

To examine the role of Gal-1-induced FN and LM-5 in cell motility, we assessed whether Gal-1-related FN and LM-5 was secreted into the medium. As shown in Figures 7a and b, Gal-1 stimulated FN (700 ng/ml increase compared with the control; *P*<0.05) and LM-5 (62.5 ng/ml increase compared with the control; *P*<0.05) production in medium. In addition, UCB-MSCs were treated with Gal-1 (10 ng/ml), FN (700 ng/ml), LM-5 (62.5 ng/ml), the combination of FN and LM-5, and the combination of FN, LM-5, and Gal-1 (0.01 ng/ml). As shown in Figures 7c and d, all treatment increased cell motility. One of the notable points was that the combination of FN and LM-5 (148% increase compared with the control; *P*<0.05) increased cell motility more than that of FN (65% increase compared with the control; *P*<0.05) or LM-5 (38% increase compared with the control; *P*<0.05) single treatment, and the combination of FN and LM-5 had synergistic effects on cell motility (Figures 7c and d). In addition, treatment with Gal-1 (10 ng/ml), FN (700 ng/ml), LM-5 (62.5 ng/ml), the combination of FN and LM-5, and the combination of FN, LM-5, and Gal-1 (0.01 ng/ml) increased phosphorylation and expression of FAK, Akt, and Erk (Figure 7e).

## Discussion

Our data demonstrate that Gal-1 stimulated motility that was mediated by downregulation of smad2/3-dependent COL-3/-5, and upregulation of NF-*κ*B-dependent FN/LM-5 expression via c-Src/Cav-1 and PKC in human UCB-MSCs. Thus, our findings suggest that Gal-1 may be considered a candidate agent for inducing cell motility in cell transplantation and cell therapy. Gal-1 has a broad variety of functions and regulates biological processes, including tumorigenesis, apoptosis, immune response, and determination of cell fate in stem cells.^3, 8, 24, 26^ In addition, Gal-1 has been shown to have an important role in enhancing expression of a more vasculogenic phenotype in MSCs.^27^ Similarly, the contributions of Gal-1 to the biologic behavior of stem cells are initiated by its binding specificity for *β*-galactoside-containing glycoconjugates^2, 28^ that in turn transduce the extracellular signals into intracellular molecules that perform physiological functions. We first showed that Gal-1 at ≥0.1 ng/ml significantly increased cell motility. In addition, 10 ng/ml Gal-1 maximally increased cell motility during a 24 h incubation period. Our previous report showed that Gal-1-induced motility was inhibited by *β*-lactose that blocks the interaction between Gal and glycans.^3, 26^ These findings strongly suggest that Gal-1 plays a pivotal role in stimulating cell motility via the *β*-galactoside carbohydrate structure.

In addition, Gal-1 was not detected in human UCB-MSC culture media, although human UCB-MSCs endogenously express Gal-1. Regarding the role of intrinsic intracellular Gal-1 in UCB-MSCs, the present results showed that cell motility was decreased by siRNA silencing of Gal-1 expression. Nonetheless, endogenous Gal-1 mRNA expression was not influenced by Gal-1 treatment, suggesting that intrinsic Gal-1 may independently contribute to UCB-MSCs motility at a post-translational level. These results are further supported by a previous study in which intracellular Gal-1 induced migration and invasion of glioblastoma multiforme cells.^29^ However, given that intrinsic Gal-1 can bind to a number of ECM components, such as laminin and fibronectin,^28^ it remains possible that in the present study, preferential binding of Gal-1 to ECM components interfered with our ability to detect the basal levels of any secreted forms of intrinsic Gal-1.

We and others have suggested that multiple signaling pathways such as activation of c-Src, Cav, and PKC are rapidly stimulated in target cells through Gal-1 membrane receptors and that these pathways have been linked to discrete or allied cellular actions of Gal-1.^3, 30^ Our results shed light on the potential role of c-Src-dependent Cav-1 activation in Gal-1-induced cell motility. Cav-1 has emerged as an important regulatory molecule in signal transduction.^11, 31^ It has been postulated that tyrosine phosphorylation of Cav-1 at residue 14 could confer subsequent growth-stimulatory or invasive activity that is induced by activated c-Src.^3, 32, 33^ c-Src is localized on the plasma membrane and is often associated with lipid rafts and SC motility.^34, 35^ In this study, Gal-1 led to phosphorylation of c-Src and Cav-1 that was decreased by *β*-lactose and PP2. In addition, Gal-1-induced increases in motility decreased by M*β*-CD. PKC phosphorylation and translocation increased in response to Gal-1 but did not affect intracellular Ca^2+^ concentration ([Ca^2+^]_i_), although our and other studies showed that Gal-1 triggers phosphorylation of phospholipase C^3, 36^ that produces inositol-1,4,5-triphosphate and increases [Ca^2+^]_i_ in various cell types including stem cells.^3, 37^ This discrepancy in our results might because of differences in species, cell types, or experimental conditions. Nevertheless, our results indicated that inhibition of PKC pathways caused a decrease in Gal-1-induced motility. These observations suggest that Gal-1 induced c-Src-dependent Cav-1 phosphorylation and activated PKC, and that these signaling pathways played an important role in Gal-1-induced human UCB-MSC motility.

Previous studies have suggested that Gal-related signal molecules influence ECM proteins such as COL, FN, and LM, and regulate cell motility and attachment.^38, 39^ The present results show that Gal-1 reduced *COL-3/-5* mRNA and protein levels but increased *FN*/*LM-γ2* mRNA and FN/LM-5 protein expression that was reversed by *β*-lactose. Interestingly, alteration in Gal-1-related COL-3/-5, fibronectin, and LM-5 expression plays a contrasting role in various cells and tissues. Cancer cells secrete collagenase and then disassemble collagen molecules to induce invasion and metastasis.^40^ In contrast, collagen can inhibit cancer cells and fibroblast motility by inhibiting MMP secretion and expression in adjacent cells.^16, 41^ Thus, it is possible that Gal-1-reduced COL-3/-5 stimulates human UCB-MSC motility. Furthermore, Gal-1 increased FN protein, LM-*γ*2 mRNA, and LM-5 protein expression, the results supported by previous studies in which Gal-1-related FN/LM increased phosphorylation of epidermal growth factor receptor (EGFR) and src-dependent fibroblast growth factor receptor-1 (FGFR-1) via interaction of integrins, eventually leading to increased cell motility.^42, 43^ We observed coincident LM-*γ*2 and LM-5 expression. Processing of the LM-*γ*2 chain to the mature form decreases cell adhesion activity but increases cell migration and invasion activity of LM-5 in human cancer cells.^44^ These results suggest that the Gal-1-induced LM-*γ*2 may contain a functional domain that regulates cellular adhesion and migration as well as Gal-1-mediated induction of LM-5.

Different temporal patterns of Gal-1-related ECM protein expression are because of different signaling pathways related to transcription factor upstream molecules such as PKC, Cav, and transcription factors including smad2/3 and NF-*κ*B on *ECM* genes.^17, 18, 45, 46, 47^ Recent reports have suggested that Cav-dependent signaling inhibited smad2/3 phosphorylation, whereas PKC-dependent signaling stimulated NF-*κ*B phosphorylation involved in the regulation of ECM protein synthesis.^45, 47^ In the present study, Gal-1 decreased smad2/3 phosphorylation and increased NF-*κ*B phosphorylation that was reversed by inhibiting Cav-1 and PKC. In addition, inhibiting Cav-1 alone did not affect Gal-1-reduced smad2/3 phosphorylation, and inhibiting PKC alone did not influence Gal-1-induced NF-*κ*B phosphorylation. These results indicated that Cav-1 and PKC are parallel Gal-1-dependent signals for smad2/3 and NF-*κ*B. Furthermore, Gal-1 and *smad2/3* siRNA inhibited COL-3 and COL-5 expression, and Gal-1-induced stimulation of FN and LM-5 expression was blocked by NF-*κ*B inhibitors. These results are supported by previous studies that activated Cav inhibits smad2/3 phosphorylation that is reduced by COL expression^45, 48^ and that NF-*κ*B activation has relevance to regulate ECM proteins such as FN and LM as well as motility.^18, 45, 46^ Therefore, our results strongly suggest that Cav-1-dependent smad2/3 dephosphorylation and PKC-dependent NF-*κ*B phosphorylation play an important role in Gal-1-reduced COL-3/-5 expression and Gal-1-induced FN/LM-5 expression in human UCB-MSCs.

Our results show a role for downregulation of COL-3/-5 and upregulation of FN/LM-5 in Gal-induced cell migration and its related signal molecules, suggesting that alterations in Gal-1-related COL, FN, and LM-5 expression regulate multiple signal transductions such as Erk, focal adhesion kinase (FAK), Akt, integrins, and MMPs, and then induce cell motility.^43, 46, 49, 50, 51^ We showed here that Gal-1, *COL-3*, and/or *COL-5* siRNA treatment induced motility and increased MMP2 and IN-*β*1 expression in human UCB-MSCs. Interestingly, the combined treatment of *COL-3/-5* siRNAs was more effective on motility and related protein expression than a single treatment of siRNAs, although treatment of human UCB-MSC with Gal-1 led to maximum increased motility and related protein expression. These results are supported by a previous study showing that ECM proteins have synergistic effects on cell motility^11, 14^ and that downregulation of COL-induced invasion in cancer cells through increased MMP and IN-*β*1 expression reduced E-cadherin expression.^14, 52^ In addition, we observed that treatment with Gal-1, FN, and/or LM-5 stimulated motility and then increased FAK, Akt, and Erk phosphorylation/expression. These results are further supported by other studies using embryonic stem cells and MSCs, suggesting that FN and LM-5 mediate the regulatory effects on various downstream targets via the FAK, Akt, PDGFR-*β*, and Erk signaling pathways.^11, 13, 14^

Collectively, our results suggest that Gal-1-reduced COL-3/-5 or Gal-1-induced FN/LM-5 stimulated other multiple signaling molecules that contributed to increase human UCB-MSC motility. Recent emerging evidence shows that neonatal lymphocytes are a major extracellular source of Gal-1 in UCB,^53^ indicating that UCB-MSC motility could be regulated by extracellular Gal-1 produced by neonatal lymphocytes in *in vivo* microenvironments. In conclusion, highlighting signaling pathways involved in Gal-1-stimulated human UCB-MSC motility provides potential targets for strategic modulation of stem cell motility in contexts where improved tropism or transmigration could improve therapeutic performance.

## Materials and Methods

### Materials

Human UCB-MSCs (hUCB-MSCs) were obtained from MEDIPOST Co. Ltd (Seoul, Korea). Mouse ESCs were obtained from American Type Culture Collection (ES-E14TG2a; Manassas, VA, USA). Human AD-MSCs were kindly provided by Professor Kyung-Sun Kang (Seoul National University, Seoul, Korea). Fetal bovine serum (FBS) was purchased from BioWhittaker Inc. (Walkersville, MO, USA). FN and LM-5 was purchased from Abcam (Cambridge, UK). Gal-1 was obtained from the R&D Systems (Minneapolis, MN, USA). A23187, PP2, methyl-*β*-cyclodextrin, bisindolylmaleimide I, staurosporine, and mitomycin C were obtained from Sigma Chemical Company (St. Louis, MO, USA). *β*-Actin, Akt, caveolin-1, collagen 1, collagen 3, collagen 5, c-Src, Erk, FAK, fibronectin, flotillin-2, galectin-1, integrin-*β*1, laminin 5, laminA/C, NF-*κ*B, MMP2, MMP9, pan-cadherin, phospho-Akt^thr308^, phospho-Akt^ser473^, phospho-caveolin-1, phospho-c-Src, phospho-Erk, phospho-FAK, phospho-NF-*κ*B (p65), PKC, PKC*α*, PKC*β*, PKC*γ*, PKC*δ*, PKC*ɛ*, PKC*θ*, PKC*ζ*, and smad antibodies were purchased from Santa Cruz Biotechnology (Santa Cruz, CA, USA). Phospho-PKC and phospho-smad2/3 antibodies were purchased from Cell Signaling (Beverly, MA, USA). Horseradish peroxidase (HRP)-conjugated goat anti-rabbit and goat anti-mouse immunoglobulin G (IgG) were purchased from Jackson Immunoresearch (West Grove, PA, USA). All other reagents were of the highest purity commercially available and were used as received.

### Culture of human UCB-MSCs

Human UCB-MSCs were cultured without a feeder layer in *α*-minimum essential medium (*α*-MEM; Thermo, Tewksbury, MA, USA), 1% penicillin, 1% streptomycin, and 10% FBS. For each experiment, cells were grown in 6- and 12-well plates, and in 35, 60, or 100-mm diameter culture dishes in an incubator maintained at 37°C with 5% CO_2_. The medium was replaced with serum-free *α*-MEM at least 24 h before experiments. Following incubation, the cells were washed twice with phosphate-buffered saline (PBS) and then maintained in a serum-free *α*-MEM including all supplements and indicated agents.

### RNA isolation and reverse transcription-PCR (RT-PCR)

Total RNA was extracted from human UCB-MSCs using the RNeasy Plus Mini Kit (Qiagen, Valencia, CA, USA). RT was carried out with 3 *μ*g of RNA using a Maxime RT premix kit (iNtRON Biotechnology, Sungnam, Korea). The complementary DNA (cDNA; 5 *μ*l) for *COL* isotypes, *LM* isotypes, and *FN* cDNAs were amplified using the primers described in Supplementary Table 1.

### Real-time PCR

The real-time quantification of *COL* isotypes, *LM* isotypes, and *FN* cDNAs was performed using a Rotor-Gene 6000 real-time thermal cycling system (Corbett Research, Mortlake, NSW, Australia) with a QuantiMix SYBR Kit (PhileKorea Technology, Daejeon, Korea), 20 *μ*l reaction mixture contained 200 ng cDNA, 0.5 *μ*M of each primer, enzymes, and fluorescent dyes. The data were collected during the extension step and analyzed using the manufacturer's software. To verify the specificity and identity of PCR products, the amplification cycles were followed by a high-resolution melting cycle from 65 to 99°C at a rate of 0.1 °C/2 s. When the melting temperature (Tm) was reached, double-stranded DNA was denatured and the SYBR was released that caused a dramatic decrease in fluorescence intensity. The rate of this change was determined by plotting the derivative of the fluorescence relative to the temperature (dF/dT) *versus* temperature by data analysis software of the real-time PCR instrument. The temperature at which a peak occurred on the plot corresponded to the Tm of the DNA duplex. *β*-Actin was used as an endogenous control and a normalization control was used as a defined calibrator.

### Boyden chamber migration assay

Gal-1-induced migration was measured by a modified Boyden chamber assay, using a 48-well micro-chemotaxis chamber (NeuroProbe, Baltimore, MD, USA) with 8 *μ*m pore size polycarbonate filters (Whatman Biometra, Gottingen, Germany) as described previously.^54^ The lower wells were filled with serum-free *α*-MEM and covered by the chemotaxis filter. The upper wells received 1 × 10^4^ cells and Gal-1 (0–100 ng/ml) in 50 ml *α*-MEM. After 24 h of incubation, the filter was carefully removed and nonmigrating cells on the upper side were eliminated by rinsing with cold PBS and scraping of the surface with a rubber wiper. Cells that had migrated into the solution on the lower side of the filter were fixed with 4% formaldehyde and stained with Giemsa. The number of migrating cells in control and stimulated wells was counted at × 100 magnification. Results were expressed as a percent of control, determined as the average number of migrated cells in stimulated wells divided by the average number of migrating cells in control wells.

### Wound healing migration assay

Human UCB-MSCs were seeded at 4 × 10^4^ cells on low 35-mm dishes with culture inserts (Ibidi, Martinsried, Germany)^35, 55^ and incubated until the cell reached around 100% confluence in serum-containing medium. After serum starvation for 24 h, inserts were removed with sterile forceps to create a wound field of ∼500 *μ*m. The cells were incubated for an additional 24 h in Gal-1 (10 ng/ml) and visualized with an Olympus FluoView 300 confocal microscope (Melville, NY, USA) with × 400 objective.

### Oirs cell migration assay

Human UCB-MSCs were seeded at 3 × 10^2^ cells/100 *μ*l in Oirs well (Platypus Technologies) and incubated for 24 h to permit cell adhesion. Inserts were carefully removed when the cell reached around 70% confluence, and the wells were gently washed with culture medium. Cells were then incubated with gal-1 (10 ng/ml) and serum-free medium. Cell motility was observed microscopically after 24 h. Cell populations in end point assays were stained with 5 *μ*M calcein AM for 30 min. Migrated cells were quantified through measurement of fluorescence signals by using a microplate reader at excitation and emission wavelengths of 485 and 515 nm, respectively.^56^

### Immunofluorescence microscopy

Human UCB-MSCs were plated onto coverslips (thickness: no 1, size: 18 mm) (Thermo Fisher Scientific, Rockford, IL, USA), serum starved for 24 h, and then treated for 1 h with lipid raft disruptor M*β*-CD (10 mM). Cells were fixed with 3.5% paraformaldehyde in PBS, permeabilized for 10 min with 0.1% (vol/vol) Triton X-100, and washed three times for 10 min each with PBS. Cells preincubated with 10% bovine serum albumin (BSA; Sigma-Aldrich, St. Louis, MO, USA) in PBS for 20 min to decrease nonspecific antibody binding were incubated for 60 min with a 1 : 100 dilution of primary antibody (anti-Cav-1 antibody) in a solution containing 1% (v/v) BSA in PBS, and washed three times for 10 min each with PBS. Cells were then incubated with 1% (v/v) BSA for 5 min, incubated for 60 min with anti-rabbit IgM-FITC (green) antibody, counterstained with propidium iodide (PI) in PBS containing 1% (v/v) BSA, and washed three times for 10  min each with PBS. Samples were mounted on slides and visualized with an Olympus FluoView 300 confocal microscope with × 400 objective.

### siRNA transfection

Human UCB-MSCs were grown until 75% of the surface of the plate was covered, after which they were transfected for 24 h with either a siRNA specific for *COL-3*, *COL-5*, *smad2/3* (200 pmol/l; GenePharma, Shanghai, China) or a *non-targeting* siRNA as a negative control (200 pmol/l; GenePharma) with Hyperfectamine (Qiagen) according to the manufacturer's instructions. The sequences used are described in Supplementary Table 2, and determined each siRNA efficacy and effect of basal level, respectively (Supplementary Data 5).

### Western blot analysis

Cells were harvested, washed twice with PBS, and lysed with buffer (20 mM Tris (pH 7.5), 1 mM ethylenediaminetetraacetic acid (EDTA), 1 mM ethylene glycol tetraacetic acid (EGTA), 1% Triton X-100, 1 mg/ml aprotinin, and 1 mM phenylmethylsulfonylfluoride (PMSF)) for 30 min on ice. The lysates were then cleared by centrifugation (22 250 × *g* at 4°C for 30 min). Protein concentration was determined by the Bradford method.^57^ Equal amounts of protein (20 *μ*g) were resolved by 10% sodium dodecyl sulfate polyacrylamide gel electrophoresis (SDS-PAGE) and transferred to a polyvinylidene fluoride (PVDF) membrane. The membranes were washed with Tris-buffered saline with Tween-20 (TBST) solution (10 mM Tris-HCl (pH 7.6), 150 mM NaCl, and 0.05% Tween-20), and blocked with 5% skimmed milk for 1 h. An appropriate primary antibody was incubated at 4°C overnight. The membrane was then washed and detected with a HRP-conjugated secondary antibody. The bands were visualized by enhanced chemiluminescence (Amersham Pharmacia Biotech Inc., Buckinghamshire, UK).

### Enzyme-linked immunosorbent assay (ELISA) for FN and LM-5

Concentrations of FN and LM secreted in cell culture media were quantified by competitive inhibition ELISA using QuantiMatrix Human Fibronectin ELISA Kit and QuantiMatrix Human Laminin ELISA Kit (Chemicon International Inc., Temecula, CA, USA) according to the manufacturer's instructions. The human UCB-MSC culture media were replaced with serum-free α-MEM at least 24 h before the assays, and the media supernatants were collected by centrifugation. A 100 *μ*l supernatant was loaded into a 96-well plate and read at 450 nm using spectrophotometer (Victor3; Perkin-Elmer, Waltham, MA, USA). Results were expressed as the mean concentration of triplicate cultures. Values were converted from absolute counts to a percentage of the control to allow for comparison between experimental groups.

### TCA precipitation

Filtered culture supernatants were mixed with trichloroacetic acid (TCA) to a final concentration of 30% (w/v) and were incubated on ice for 30 min or stirred overnight at 4°C. Samples were centrifuged at 10 000 × *g* for 20 min. Pellets were washed with ice-cold 96% ethanol (v/v) and acetone and were air-dried.

### Measurement of calcium influx

Changes in intracellular calcium concentrations were monitored using Fluo-3-AM that had initially been dissolved in dimethylsulfoxide (DMSO). Cells in 35 mm-diameter culture dished were rinsed with a Bath Solution (140 mM NaCl, 5 mM KCl, 1 mM CaCl_2_, 0.5 mM MgCl_2_, 10 mM glucose, 5.5 mM 4-(2-hydroxyethyl)-1-piperazineethanesulfonic acid (HEPES; pH 7.4)) and were then incubated in a Bath Solution containing 3 *μ*M Fluo-3-AM for 40 min, rinsed, mounted on a perfusion chamber, and scanned at 1 s intervals using Olympus FluoView 300 confocal microscope with × 300 objective. The fluorescence was produced by excitation at 488 nm and the emitted light was observed at 515 nm. All analyses of calcium influx were processed in a single cell, and the results were expressed as the fluorescent intensity (F/F_0_%, arbitrary unit, where F is fluorescence captured at a particular time and F_0_ is initial fluorescence image captured).

### Subcellular fractionation

Harvested cell pellets were mixed with buffer 1 (250 mM sucrose, 50 mM Tris-HCl, 5 mM MgCl_2_) in the presence of protease inhibitor cocktail (PIERCE, Rockford, IL, USA) and incubated for 10 min on an end-over-end shaker and centrifuged at 1000 × *g* for 10 min. The supernatants with cytosolic protein were transferred to iced tubes. The pellet was suspended in buffer 2 (1 M sucrose, 50 mM Tris-HCl, 5 mM MgCl_2_) for 30 min and centrifuged at 6000 × *g* for 10 min and the supernatants containing membrane proteins were transferred to new tubes. The remaining pellet was suspended in buffer 3 (20 mM Tris-HCl, 0.4 M NaCl, 15% glycerol, 1.5% Triton X-100) with protease inhibitor cocktail and incubated for 10 min on an end-over-end shaker. After centrifugation at 6800 × *g* for 10 min, the supernatants was collected and designed as the nuclear proteins.

### Detergent-free purification of caveolin-rich membrane fraction

Cav-enriched membrane fractions were prepared as described previously.^58^ Cells were washed twice with ice-cold PBS, scraped into 2 ml of 500 mM sodium carbonate (pH 11.0), transferred to a plastic tube, and homogenized with a Sonicator 250 apparatus (Branson Ultrasonic, Danbury, CT, USA) using three 20-s bursts. The homogenate was adjusted to 45% sucrose by the addition of 2 ml 90% sucrose prepared in 2-(N-morpholino) ethanesulfonic acid (MES)-buffered solution consisting of 25 mM MES-buffer solution (pH 6.5) and 0.15 M NaCl and placed at the bottom of an ultracentrifuge tube. A 5–35% discontinuous sucrose gradient was formed (4 ml each of 5 and 35% sucrose, both in MES-buffer solution containing 250 mM sodium carbonate) and centrifuged at 40 000 × *g* for 20 h in a Beckman SW41 Rotor (Beckman Coulter, Fullerton, CA, USA). Twelve fractions were collected and analyzed by 12% SDS-PAGE.

### Statistical analysis

Results are expressed as means±S.E. All experiments were analyzed by ANOVA, followed in some cases by a comparison of treatment means with the control using the Bonferroni–Dunn test. Differences were considered statistically significant at *P*<0.05.

## Abbreviations

Cav: caveolin
cDNA: complementary DNA
COL: collagen
ECM: extracellular matrix
EDTA: ethylenediaminetetraacetic acid
EGFR: epidermal growth factor receptor
EGTA: ethylene glycol tetraacetic acid
ESC: embryonic stem cell
FAK: focal adhesion kinase
FBS: fetal bovine serum
FGFR: fibroblast growth factor receptor
FITC: fluorescein isothiocyanate
FN: fibronectin
Gal: galectin
HEPES: 4-(2-hydroxyethyl)-1-piperazineethanesulfonic acid
Ig: immunoglobulin
IN: integrin
LM: laminin
*α*-MEM: *α*-minimum essential medium
M*β*-CD: methyl-*β*-cyclodextrin
MMP: matrix metalloproteinase
NF-*κ*B: nuclear factor *κ*-light-chain-enhancer of activated B cells
NSC: neural stem cell
Pan-cad: pan-cadherin
PBS: phosphate-buffered saline
PDGFR: platelet-derived growth factor receptor
PI: propidium iodide
PKC: protein kinase C
PVDF: polyvinylidene fluoride
RT-PCR: reverse transcription-PCR
SDS-PAGE: sodium dodecyl sulfate-polyacrylamide gel electrophoresis
siRNA: small interfering RNA
TBST: Tris-buffered saline with Tween-20
TCA: trichloroacetic acid
Tm: melting temperature
UCB-MSC: umbilical cord blood-derived mesenchymal stem cell
