# Supplementary material for: Galectin-1 stimulates motility of human umbilical cord blood-derived mesenchymal stem cells by downregulation of smad2/3-dependent collagen 3/5 and upregulation of NF-κB-dependent fibronectin/laminin 5 expression
Source: Cell Death Dis. 2014 Feb 6;5(2):e1049–. doi: 10.1038/cddis.2014.3 (PMC3944255; doi:10.1038/cddis.2014.3)
Supplement: Supplementary Data 1 [file cddis20143x1.doc]

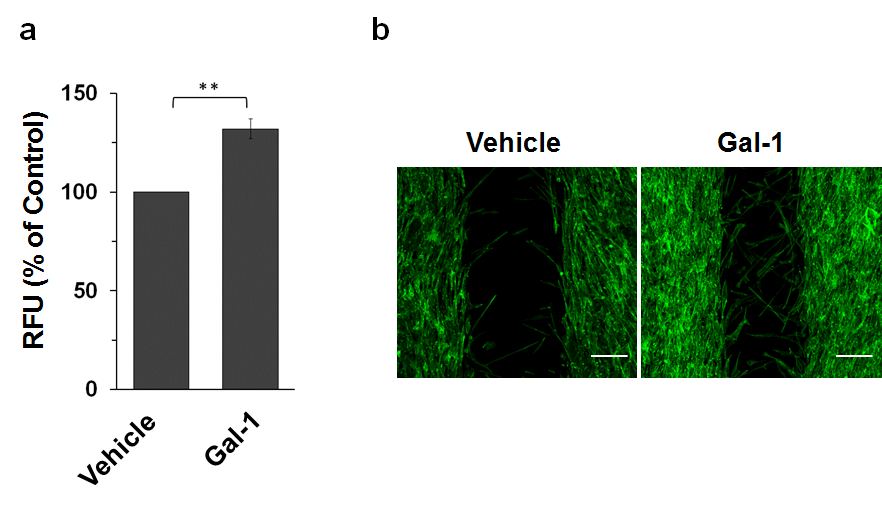


**Supplemental Data 1.** **Effect of** **Gal-1 on human adipose-derived mesenchymal stem cells (AD-MSCs) motility.** (a) OrisTM cell migration assay. AD-MSCs were treated with Gal-1 (10 ng/ml) for 24 h and stained with calcein AM. Fluorescence in the analytical zone was quantified with a plate reader. Error bars represent the means + SE from three independent experiments. **, *P* < 0.01 vs Vehicle. (b) Wound-healing assay. AD-MSCs treated with Gal-1 (10 ng/ml) for 24 h were fixed and labeled with phalloidin-AlexaFluor 488 (green) to identify the migrating cells**.** n=3. Scale bars represent 100 μm (magnification, ×100).
