# Supplementary material for: Galectin-1 stimulates motility of human umbilical cord blood-derived mesenchymal stem cells by downregulation of smad2/3-dependent collagen 3/5 and upregulation of NF-κB-dependent fibronectin/laminin 5 expression
Source: Cell Death Dis. 2014 Feb 6;5(2):e1049–. doi: 10.1038/cddis.2014.3 (PMC3944255; doi:10.1038/cddis.2014.3)
Supplement: Supplementary Data 2 [file cddis20143x2.doc]

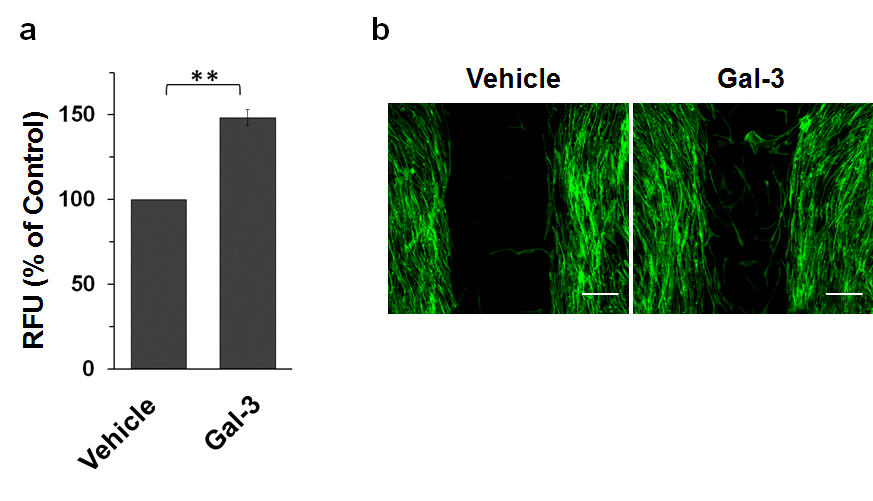


**Supplemental Data 2. Effect of Gal-3 on UCB-MSCs motility.** (a) OrisTM cell migration assay**.** UCB-MSCs were treated with Gal-3 (10 ng/ml) for 24 h and stained with calcein AM. Fluorescence in the analytical zone was quantified with a plate reader Error bars represent the means + SE from three independent experiments. **, P < 0.01 vs Vehicle. (b) Wound-healing assay. UCB-MSCs treated with Gal-3 (10 ng/ml) for 24 h were fixed and labeled with phalloidin-AlexaFluor 488 (green) to identify the migrating cells. n=3. Scale bars represent 100 μm (magnification, ×100).
