# Supplementary material for: Galectin-1 stimulates motility of human umbilical cord blood-derived mesenchymal stem cells by downregulation of smad2/3-dependent collagen 3/5 and upregulation of NF-κB-dependent fibronectin/laminin 5 expression
Source: Cell Death Dis. 2014 Feb 6;5(2):e1049–. doi: 10.1038/cddis.2014.3 (PMC3944255; doi:10.1038/cddis.2014.3)
Supplement: Supplementary Data 3 [file cddis20143x3.doc]

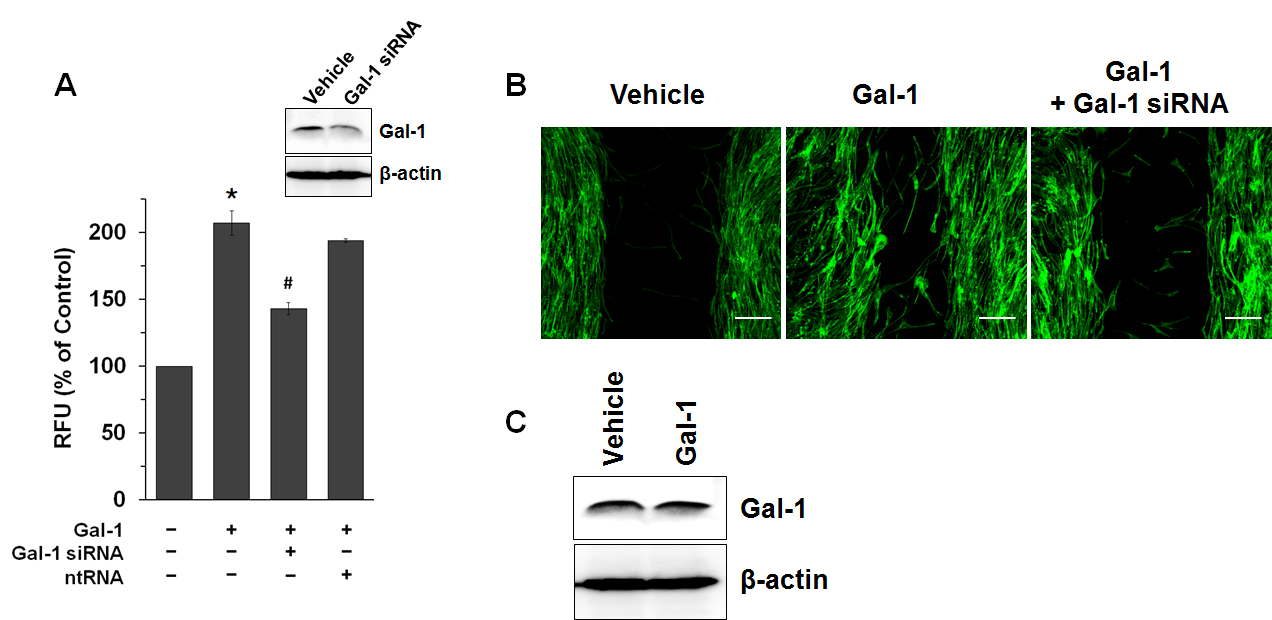


**Supplemental Data 3.** **Effect of** **intracellular** **Gal-1 on UCB-MSCs** **motility.** UCB-MSCs transfected with Gal-1 siRNA were treated with Gal-1 (10 ng/ml) for 24 h. (a) OrisTM cell migration assay. Cells were stained with calcein AM. Fluorescence in the analytical zone was quantified with a plate reader Error bars represent the means + SE from three independent experiments. *, *P <* 0.05 vs Vehicle. ^#^, *P* < 0.05 vs Gal-1treatment. The inset shows the efficacy of Gal-1 knockdown (67%) determined by Western blot. (b) Wound-healing assay. Cells were fixed and labeled with phalloidin-AlexaFluor 488 (green) to identify the migrating cells**.** n=3. Scale bars represent 100 μm (magnification, ×100). (c) Expression of Gal-1 in UCB-MSCs treated with Gal-1 was determined. n = 3.
