# Supplementary material for: Galectin-1 stimulates motility of human umbilical cord blood-derived mesenchymal stem cells by downregulation of smad2/3-dependent collagen 3/5 and upregulation of NF-κB-dependent fibronectin/laminin 5 expression
Source: Cell Death Dis. 2014 Feb 6;5(2):e1049–. doi: 10.1038/cddis.2014.3 (PMC3944255; doi:10.1038/cddis.2014.3)
Supplement: Supplementary Data 4 [file cddis20143x4.doc]

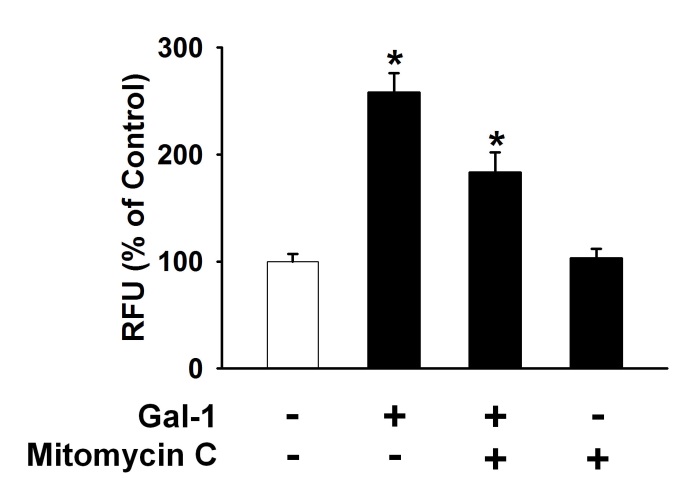


**Supplemental Data 4. Effect of mitomycin C in Gal-1-induced cell migration.** Oris cell migration assay. Cells were pretreated with mitomycin C for 30 min prior to 10 ng/ml Gal-1 treatment for 24 h, and stained with calcein AM (5 μM). Fluorescence in the analytical zone was quantified with a plate reader. Data represent means ± SE of five independent experiments with triplicate dishes. **P* < 0.05 vs. control.
