# Supplementary material for: Galectin-1 stimulates motility of human umbilical cord blood-derived mesenchymal stem cells by downregulation of smad2/3-dependent collagen 3/5 and upregulation of NF-κB-dependent fibronectin/laminin 5 expression
Source: Cell Death Dis. 2014 Feb 6;5(2):e1049–. doi: 10.1038/cddis.2014.3 (PMC3944255; doi:10.1038/cddis.2014.3)
Supplement: Supplementary Data 5 [file cddis20143x5.doc]

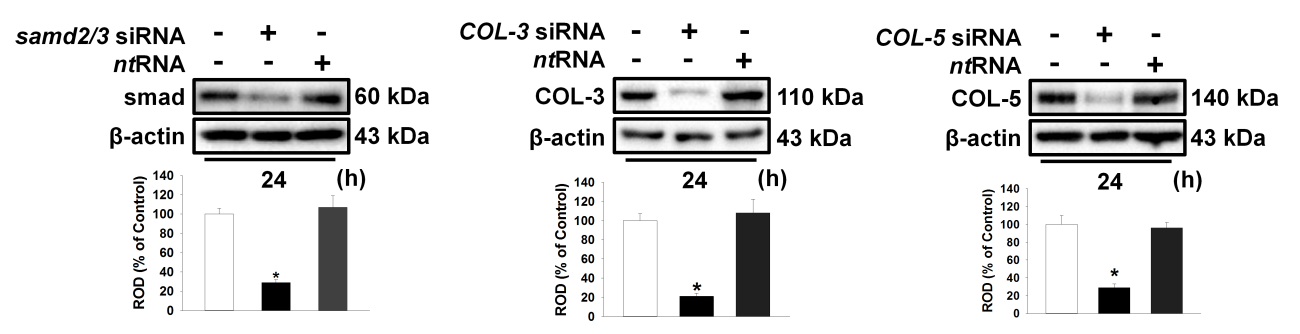


**Supplemental Data 5. Effect of siRNA on target proteins.** Cells were transfected for 24 h with either *smad2/3*-, *COL-3*, and *COL-5* siRNA (200 pmol/L) or *non-targeting* control siRNA (200 pmol/L) using Hyperfectamine. Smad, COL-3, and COL-5 expression was analyzed using Western blot. Each of the examples shown is representative of five independent experiments. **P* < 0.05 vs. control.
