# Supplementary material for: Galectin-1 stimulates motility of human umbilical cord blood-derived mesenchymal stem cells by downregulation of smad2/3-dependent collagen 3/5 and upregulation of NF-κB-dependent fibronectin/laminin 5 expression
Source: Cell Death Dis. 2014 Feb 6;5(2):e1049–. doi: 10.1038/cddis.2014.3 (PMC3944255; doi:10.1038/cddis.2014.3)
Supplement: Supplementary Table 1 [file cddis20143x6.doc]

**Supplemental table 1. Primers used for polymerase chain reaction**

| **Gene** | **Identification** | **Primer sequence, 5’–3’** | **Annealing**  **Tm. (**ºC**)** | **Size**  **(bp)** |
| --- | --- | --- | --- | --- |
| ***COL1A2 (COL-1)*** | Sense  Antisense | GGTGGTGGTTATGACTTTGG  GTTCTTGGCTGGGATGTTTT | 56 | 371 |
| ***COL2A1 (COL-2)*** | Sense  Antisense | CAACACTGCCAACGTCCAGAT  CTGCTTCGTCCAGATAGGCAAT | 56 | 156 |
| ***COL3A1 (COL-3)*** | Sense  Antisense | GCTCTGCTTCATCCCACTATTA  TGCGAGTCCTCCTACTGCTAC | 56 | 471 |
| ***COL4A1 (COL-4)*** | Sense  Antisense | GCCCATGGTCAGGACTTG  AAGGGCATGGTGCTGAACT | 56 | 61 |
| ***COL5A1 (COL-5*** | Sense  Antisense | GTCCCCCTCAAACACTTCCT  TCTCAGCGTCCACAAGAAAA | 60 | 154 |
| ***FN1***  ***(FN)*** | Sense  Antisense | TCTGTAGGCCGTTGGAAGGAAG  AGGCGCTGTTGTTTGTGAAGTAGA | 56 | 486 |
| ***LAMA1 (LM-α1)*)** | Sense  Antisense | AAGTGTGAAGAATGTGAGGATGGG  CACTGAGGACCAAAGACATTTTCCT | 56 | 317 |
| ***LAMA2 (LM-α2)*** | Sense  Antisense | AAATGTACAGAGTGCAGTCGAGGTCA  CAGTGGATGCCTTCCACATTCACCTT | 60 | 170 |
| ***LAMA3 (LM-α3)*** | Sense  Antisense | CACTGTGAACGCTGCCAGGAGGGCTA  CAGCTACCTCCGAATTTCTGGGGATT | 60 | 212 |
| ***LAMA4 (LM-α4)*** | Sense  Antisense | CACTGTGAAAAGTGTCTGGATGGT  CAGGTGCTTCCAATGAGGAAGGGG | 57 | 227 |
| ***LAMA5 (LM α5)*** | Sense  Antisense | GAAGCTGGCTCTTGTCATCC  GCATAATCCAGGCCAAAGAA | 56 | 299 |
| ***LAMB1 (LM-β1)*** | Sense  Antisense | AACTGTGAGCAGTGCAAGCCGTTT  CAACCAAATGGATCTTCACTGCTT | 55 | 248 |
| ***LAMB2 (LM-β2)*** | Sense  Antisense | CAGTGTGAGCTCTGTCGG CAAGGAGTGCTCCCAGGC | 60 | 299 |
| ***LAMB3 (LM-β3)*** | Sense  Antisense | CTGCTTCTGTCACGGCCAT  CACGCACACCGGGTAGC | 58 | 991 |
| ***LAMC1 (LM-γ1*)** | Sense  Antisense | ACGGCTACTTTGGAGACCCT  GTCCAAACCCAAAGTGGTTG | 56 | 504 |
| ***LAMC2 (LM-γ2)*** | Sense  Antisense | AAAGCCACGTTGAGTCAGCC  TCTTCCACCTGAAAGGACTG | 58 | 313 |
| ***LAMC3***  ***(LM-γ3)*** | Sense  Antisense | GCGTCCACTGCCCAGTTCCAGGTG  AATCTCCACCCAGGAGGCTGGCGG | 60 | 549 |
| ***beta ACTB (β-Actin)*** | Sense  Antisense | AACCGCGAGAAGATGACC  AGCAGCCGTGGCCATCTC | 55 | 351 |
