# Supplementary material for: Galectin-1 stimulates motility of human umbilical cord blood-derived mesenchymal stem cells by downregulation of smad2/3-dependent collagen 3/5 and upregulation of NF-κB-dependent fibronectin/laminin 5 expression
Source: Cell Death Dis. 2014 Feb 6;5(2):e1049–. doi: 10.1038/cddis.2014.3 (PMC3944255; doi:10.1038/cddis.2014.3)
Supplement: Supplementary Table 2 [file cddis20143x7.doc]

**Supplemental Table 2. siRNA sequence for transfection**

| **Gene** |  | | **Sequence, 5’–3’** | |
| --- | --- | --- | --- | --- |
| ***COL-3A1***  ***COL-5A1***  ***Smad2***  ***Smad3*** | **Sense**  **Antisense**  **Sense**  **Antisense**  **Sense**  **Antisense**  **Sense**  **Antisense**  **Sense**  **Antisense**  **Sense**  **Antisense**  **Sense**  **Antisense**  **Sense**  **Antisense**  **Sense**  **Antisense**  **Sense**  **Antisense**  **Sense**  **Antisense**  **Sense**  **Antisense**  **Sense**  **Antisense**  **Sense**  **Antisense**  **Sense**  **Antisense**  **Sense**  **Antisense**  **Sense**  **Antisense** | | **GCUGAAGGAAAUAGCAAAUTT**  **AUUUGCUAUUUCCUUCAGCTT**  **GUCUGUUAAUGGACAAAUATT**  **UAUUUGUCCAUUAACAGACTT**  **CGGUCCUAAAGGAAAUGAUTT**  **AUCAUUUCCUUUAGGACCGTT**  **GAUGCUAUCAAGGUAUUCUTT**  **AGAAUACCUUGAUAGCAUCTT**  **CGGGCAGCUUAUGAUUACUTT**  **AGUAAUCAUAAGCUGCCCGTT**  **GGUGAAGCGUCACAGAAAUTT**  **AUUUCUGUGACGCUUCACCTT**  **GGGAUUCCUUCAAGGUUUATT**  **UAAACCUUGAAGGAAUCCCTT**  **GGGAUUCCUUCAAGGUUUATT**  **UAAACCUUGAAGGAAUCCCTT**  **GAGACCUAUUACUACGAAUTT**  **AUUCGUAGUAAUAGGUCUCTT**  **GGUGUUCGAUAGCAUAUUATT**  **UAAUAUGCUAUCGAACACCTT**  **CCCUGCAACAGUGUGUAAATT**  **UUUACACACUGUUGCAGGGTT**  **CUCCAAUGUUAACCGAAAUTT**  **AUUUCGGUUAACAUUGGAGTT**  **GACCAACAGUUGAAUCAAATT**  **UUUGAUUCAACUGUUGGUCTT**  **GCAACCUGAAGAUCUUCAATT**  **UUGAAGAUCUUCAGGUUGCTT**  **GCGUGAAUCCCUACCACUATT**  **UAGUGGUAGGGAUUCACGCTT**  **CGCAGGUUCUCCAAACCUATT**  **UAGGUUUGGAGAACCUGCGTT**  **CCGCAUGAGCUUCGUCAAATT**  **UUUGACGAAGCUCAUGCGGTT** | |
| ***Gal-1***  ***(LGALS1)***  ***Nt*** | | **Sense**  **Antisense**  **Sense**  **Antisense**  **Sense**  **Antisense** | | **GCUGCCAGAUGGAUACGAA**  **CCAGCAACCUGAAUCUCAA**  **ACGGUGACUUCAAGAUCAA**  **CUAAGAGCUUCGUGCUGAA**  **UAGCGACUAAACACAUCAA**  **UUGAUGUGUUUAGUCGCUA** |
